# 3D Printing and NIR Fluorescence Imaging Techniques for the Fabrication of Implants

**DOI:** 10.3390/ma13214819

**Published:** 2020-10-28

**Authors:** Yong Joon Suh, Tae Hyeon Lim, Hak Soo Choi, Moon Suk Kim, Sang Jin Lee, Soon Hee Kim, Chan Hum Park

**Affiliations:** 1 Department of Breast and Endocrine Surgery, Hallym University Sacred Heart Hospital, Anyang 14068, Korea; nicizm@gmail.com; 2 Nano-Bio Regenerative Medical Institute, College of Medicine, Hallym University, Chuncheon 24252, Korea; pepshe@naver.com; 3 Gordon Center for Medical Imaging, Department of Radiology, Massachusetts General Hospital and Harvard Medical School, Charlestown, MA 02114, USA; hchoi12@mgh.harvard.edu; 4 Department Molecular Science and Technology, Ajou University, Suwon 443-759, Korea; moonskim@ajou.ac.kr; 5 Wake Forest Institute for Regenerative Medicine, Wake Forest School of Medicine, Medical Center Boulevard, Winston-Salem, NC 27157, USA; sjlee@wakehealth.edu; 6 Departments of Otorhinolaryngology-Head and Neck Surgery, Chuncheon Sacred Heart Hospital, School of Medicine, Hallym University, Chuncheon 24252, Korea

**Keywords:** 3D printing, implants, fabrication, bioprinting, near-infrared fluorescence imaging

## Abstract

Three-dimensional (3D) printing technology holds great potential to fabricate complex constructs in the field of regenerative medicine. Researchers in the surgical fields have used 3D printing techniques and their associated biomaterials for education, training, consultation, organ transplantation, plastic surgery, surgical planning, dentures, and more. In addition, the universal utilization of 3D printing techniques enables researchers to exploit different types of hardware and software in, for example, the surgical fields. To realize the 3D-printed structures to implant them in the body and tissue regeneration, it is important to understand 3D printing technology and its enabling technologies. This paper concisely reviews 3D printing techniques in terms of hardware, software, and materials with a focus on surgery. In addition, it reviews bioprinting technology and a non-invasive monitoring method using near-infrared (NIR) fluorescence, with special attention to the 3D-bioprinted tissue constructs. NIR fluorescence imaging applied to 3D printing technology can play a significant role in monitoring the therapeutic efficacy of 3D structures for clinical implants. Consequently, these techniques can provide individually customized products and improve the treatment outcome of surgeries.

## 1. Introduction

Recently, three-dimensional (3D) printing techniques have been evolving rapidly. 3D printing is based on the principle of building objects by adding materials layer by layer [1]. Since the invention of a stereolithography apparatus (SLA) in 1984 by Charles W. Hullin, novel 3D printing methods and materials have been introduced [2,3]. 3D printing refers to the volumetric presswork of digital data converted from an imaginary model, whereas two-dimensional (2D) printing is typically achieved on paper [4]. These techniques have rapidly been adopted across many fields [5]. The ubiquitous presence of Internet computing and the availability of 3D printing enable the personalization of products tailored to individual needs. This progress enables engineers to develop various types of hardware and software that are beneficial in diverse areas [6]. 3D printing involves the use of various materials, such as filament, powder, resin, and paper [7]. 

Researchers in the surgical fields utilize these techniques due to their great potential [1,5,6,7,8,9]. 3D printing has significant implications in various surgical fields, such as education, training, consultation, surgical planning, organ transplantation, plastic surgery, and prosthesis [10,11,12,13,14]. Commercial manufacturers are developing low-priced, small-scale, customized products for patients [10]. For surgeons, a virtual 3D architecture from 2D presurgical images is limited by the difficult intuitive comprehension of interspatial relationships [15]. However, information retrieved from 3D-printed products can help surgeons augment sensory perception using tactile feedback. Additionally, different materials and devices are being tested for novel surgical applications. Recently, the number of reports has increased regarding the utilization of 3D printing techniques to solve the medical dilemma of the need for artificial organs owing to the shortage of transplantable organs caused by accelerated low birth rates and the aging population [5,6,16,17,18,19]. 

In addition, 3D bioprinting provides precise spatiotemporal control of bioactive substances including cells, proteins, DNA, drugs, and growth factors, making them more effective in tissue formation for patient-specific treatment [20]. 3D bioprinting has the potential to fabricate large structures; however, it is difficult to image the resulting structures with superficial imaging modalities. The imaging of engineered structures fabricated with a thickness of millimeter (mm) or more will be important for surgically implanting 3D printed structures. The 3D imaging of engineered tissues is of great importance in obtaining information on structures as well as cells seeded in structures by printing. After transplantation, the implant is subjected to a number of fluctuations in vivo environments such as temperature, fluid flow, and transplant depth. Therefore, it is recommended to monitor the 3D structure in a non-invasive manner in real time. Different imaging modalities have been used to monitor non-invasively implants in vivo. Near-infrared (NIR) fluorescence that presents minimal background autofluorescence, reduced light scattering, and low tissue absorption can be a safe and cost-effective imaging source with a high resolution and high sensitivity [21].

This paper concisely reviews 3D printing techniques based on surgeons’ perspectives, classified into hardware, software, materials, and bioprinting. To achieve successful tissue regeneration using 3D-printed constructs, it is important to understand its enabling technologies as well as 3D printing technology. We also summarize the studies on NIR fluorescence imaging for the non-invasive monitoring of implants. Finally, we present our concluding remarks.

## 2. Hardware in 3D Printing Techniques

**Figure 1:**
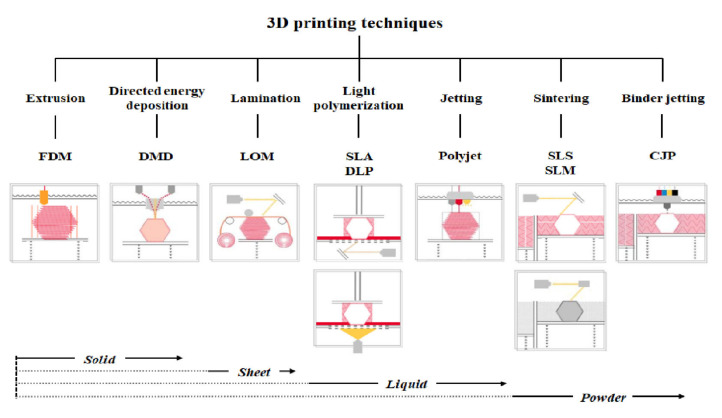
Three-dimensional printers classified according to type and material.

3D printer hardware can be categorized into seven types according to their discriminative process, although innovative products are under development [3]. These types are extrusion, direct energy deposition, sheet lamination, light polymerization, jetting, sintering, and binder jetting. Representative 3D printers include fused deposition modeling (FDM) for extrusion, direct metal deposition (DMD) for direct energy deposition, laminated object manufacturing (LOM) for lamination, SLA and digital light processing (DLP) for light polymerization, color jet 3D printing (CJP) for binder jetting, polyjet for jetting, and selective laser sintering (SLS) and selective laser melting (SLM) for sintering (Figure 1) [22]. 

**Figure 2:**
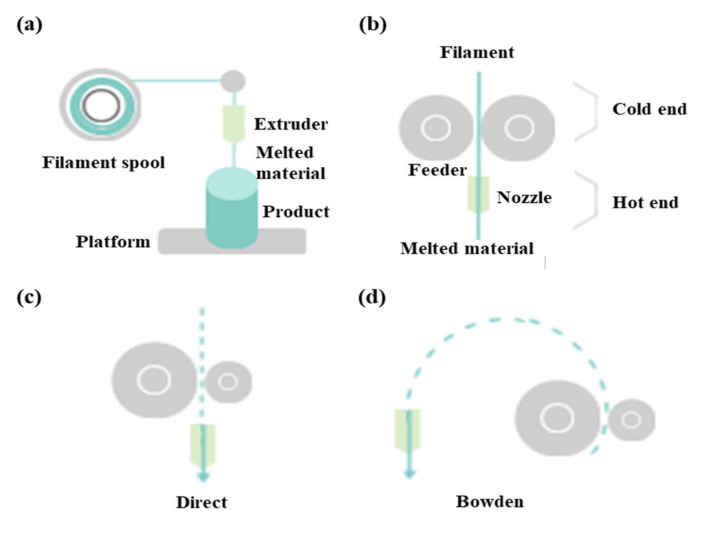
Differences between direct extruder and Bowden extruder in fused deposition modeling: (**a**) printing filament through an extruder, (**b**) distinction between cold and hot ends, (**c**) direct extruder, (**d**) Bowden extruder three-dimensional printers classified according to type and material.

FDM as a universal modeling system in surgery involves building an extruded viscous fluid layer by layer after a heated nozzle melts a solid material [9,23,24]. FDM is cheaper and easier compared with other types. Various materials can be printed using FDM. However, the printout surface is rough despite the low output speed. Therefore, FDM necessitates a burdensome post-printing process. The extruder is the salient part of FDM for printing thermoplastic filaments (Figure 2a). This extruder comprises cold and hot ends (Figure 2b). The cold end feeds the filament to the hot end, which extrudes the filament melted by the extruder nozzle. The union of cold and hot ends moves together in a direct extruder, which is the primary and original extruder in FDM (Figure 2c). In a direct extruder, wheels can push a filament continuously through a nozzle because of the short distance between the cold and hot ends. Furthermore, the filament can easily be replaced. However, this heavy structure moves unevenly, which adversely affects the output. Additionally, undesired effects can occur owing to a change in direction. Therefore, FDM should be equipped with a heavy direct extruder and allow the end structures to move slowly. Hence, the Bowden extruder, which separates a filament feeder from a nozzle, was developed. In this extruder, a filament is supplied through a tube (Figure 2d). The Bowden extruder affords vibration control and fast printing because only the hot end moves. Nonetheless, the long distance between the feeder and nozzle renders it difficult to supply the filament. While the filament passes through the tube from feeder to the nozzle, the elasticity of the tube hampers the desired advancement of the filament. Hence, flexible polylactic acid (PLA) is not used in the Bowden extruder, as it can cause the breakage or deformation of the extruder in the long distance between the feeder and nozzle. 

In DMD, heat from the laser, plasma arc, and electron beam melts materials simultaneously as the material (typically wire or metallic powder) is deposited. Furthermore, a vacuum environment is necessary to prevent electrons from interacting with molecules in the air. If this process is repeated, layers are solidified and yield an object. Additionally, an LOM technique cuts resin film or paper into certain shapes and then glues them together [24,25]. Post-processing required for this technique includes mechanical grinding, drilling, and machining. 

The SLA of vat photopolymerization has been used for the longest in 3D printing. SLAs comprise a vat containing resin with a platform at the bottom. Ultraviolet (UV) laser selectively cures the resin layer by layer. The completely cured layer ascends (or descends) and is covered uniformly by resin in the vat. After printing is completed, a secondary curing may be required depending on the resin [9,24]. A photocurable liquid resin is also used in DLP, but the related process differs slightly from that of SLAs. In DLP, an entire layer of resin is used using a UV lamp and a dimming device similar to an image projector, whereas the UV laser moves linearly in SLAs [26]. Compared with SLAs, DLP is faster and cheaper. The photopolymerization performed in these techniques enables exquisite designs and rapid printing with low noise. The support structure can be removed easily during post-processing. Although a limited amount of material is used in photopolymerization, the material and bulky equipment required are expensive. More importantly, the resin should be handled with care. Although the cured printout is not harmful, masks and gloves are necessary under the circumstances of patent air ventilation because liquid resin on a printout surface is toxic [9].

The CJP of binder jetting spurts colored liquid adhesives and hardens fine powder [26]. Although CJP occurs quickly, the printout is brittle, and its strength depends on the adhesives used. The bulky equipment used in CJP is expensive and not suitable for personal purposes. Meanwhile, a polyjet combines the techniques of SLAs and CJP, where a UV lamp immediately cures the resin sprayed layer by layer from the head. Subsequently, those layers are laminated for a complete output. Various materials and colors are used more exquisitely in polyjets than in CJP, although supports are more difficult to remove. The material and equipment required in this technique are also expensive. 

In SLS, a ruler spreads powder evenly on a platform [9]. Subsequently, a laser melts and fuses the powder selectively. These processes are repeated layer by layer in SLS. SLS does not require support for printout because the unsintered power can provide support. Therefore, the surface quality is excellent, although post-processing is required. Metal materials exhibit merits in strength and speed, which can be linked to mass production. However, SLS is bulky and expensive. Additionally, protective masks and post-processing booths are recommended for SLS. Finally, SLM melts and fuses metallic powder, whereas the laser selectively sinters powdered material in SLS [9]. In SLM, the material property is improved, although more energy is consumed. 

There are difference price ranges on the market, depending on the types of 3D printer hardware. There are also a wide range of prices in the same type, depending on whether they are for consumer or industrial use. Based on the available market information as of 2020, the usual price of 3D printer hardware is as follows: FDM (USD 5000), DMD (USD 500,000), LOM (USD 15,000), SLA (USD 3500), DLP (USD 10,000), polyjet (USD 43,000), SLS (USD 10,000), SLM (USD 80,000), and CJP (USD 100,000).

## 3. Software in 3D Printing Techniques

3D printing requires prior digital conversion in software. The overall processes are composed of modeling output sculptures, slicing geometric code (G-code), and transferring G-code [27]. G-code is a programming language recognized by 3D printers. A model source refers to files containing model information of 3D shape as the output of 3D printers. The file extension of the basic model source in 3D printers is stereolithography (STL) [23]. The STL format encodes the surface geometry of 3D objects. This format represents the surface of a model as triangular meshes [26]. A basic triangular unit is referred to as a polygon or mesh [19]. The STL format utilizes triangles because they are the least-constituent polygon, and all 3D surfaces can be divided into triangles of the simplest polygon. The smaller the triangular size and the larger the triangular number, the more exquisite the object becomes, and accordingly, the capacity increases exponentially. Slicer is a software that converts 3D data into 2D data of layers that are stacked sequentially for a certain shape. A 3D printer operates based on the G-code created in the slicer [27]. Additionally, the slicer determines the approach to fill up the empty inner space of the model source, which contains only surface information. After completing all settings including temperature and output speed, slicer creates a G-code to operate the 3D printer [28]. 

**Figure 3:**
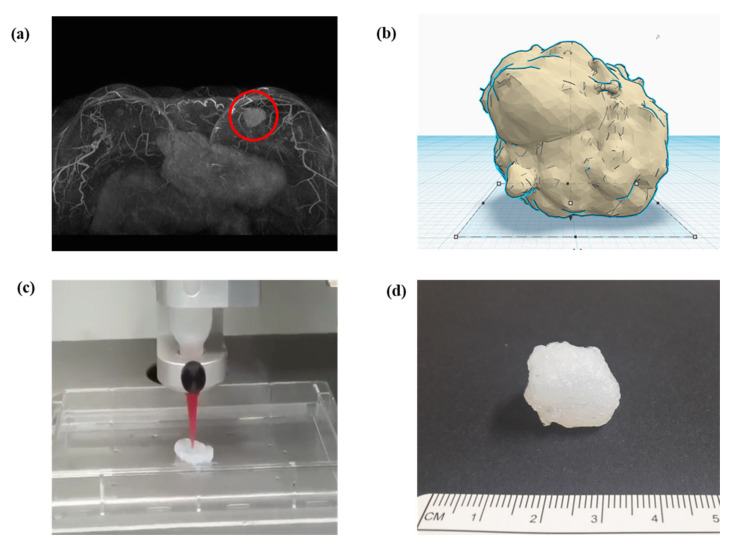
Schematic of converting a structure to (**a**) a 3D model through software and the final printout: a magnetic resonance imaging converted to stereolithography file, (**b**) modification in 3D model of stereolithography, (**c**) G-code generation in slicing, which is then delivered to a 3D printer, and(**d**) final printout after processing.

In surgeries, digital imaging and communication in medicine (DICOM) from computed tomography or magnetic resonance imaging of patients is preferentially converted to STL for 3D modeling (Figure 3a) [9,18,29,30]. Therefore, 3D design software is essential for 3D modeling (Figure 3b). An STL file can be modified adjunctively in 3D design software if necessary. Subsequently, the slicer transfers 3D data to a 3D printer for the object output, which can recognize only G-code (Figure 3c) [9,27,31]. Slicer can form crosslinks, adjust sizes, and configure various conditions, including speed, temperature, thickness, and infill density, according to the 3D printer type and material [17,23,32]. Finally, post-processing fabrication is performed to yield the final object (Figure 3d). 

The results are significantly affected by the set layer thickness. In thin layers, the output surface becomes elaborate and thin projection is post-processed readily. However, thick layers exacerbate the staircase effect on rough surfaces. The diameter of a nozzle should be set accurately for filament extrusion [23,33]. The shell means the outer wall of a 3D model. The thickness of the shell refers to the thickness of the outer wall, which combines the outermost and a few inner layers [23]. This number must be an integer multiple of the nozzle diameter. A larger thickness yields sturdier objects. The bottom and top layers are laid down before or after infill printing. The thicknesses of the bottom and top layers should be a multiple of the layer height to cover the infill adequately. Additionally, a higher infill density enables a denser and stronger lattice [23,34]. Support can be important depending on the 3D printer type. A number of variables should be considered for support, including protrusion sustenance, ease of support removal, and quality of output surface. Each slicer provides a different type of support. Moreover, before printing the first layer of the 3D model in FDM, for the adherence of the output to the platform, the software devises diverse methods such as skirt, raft, and brim. The skirt refers to borders created at regular intervals around the first layer. Raft means a layered wide plate on the platform, which places an interface between layers, and is the most reliable method to fix a printout. However, the raft requires more time and material for the printout. It can be difficult to separate this structure from the output. The brim forms a wide contact area on the platform, in which borders are repeatedly created along the border of the first layer. Compared to the raft, the brim requires a shorter time and can be easily removed.

Prontface and Cura can be used as open source host software to control 3D printers. Prontface can activate and monitor 3D printers; Cura can manipulate 3D printers diversely and create a G-code for printout. In addition, Marlin and Repetier-firmware are open source firmware software mounted on the control panel. Marlin is a popular 3D printer driver and enables the full control of the process. Repetier-firmware is more complex owing to the minute setting, which is highly compatible with its own host software.

## 4. Materials in 3D Printing Techniques

Different materials are used according to the type of 3D printer. Extrusion and direct energy deposition are based on solid materials (Figure 1). Hence, acrylonitrile butadiene styrene (ABS) and PLA are representative materials in FDM [23,26]. An ABS filament as a light synthetic material is capable of acetone fumigation in post-processing, although it tends to contract severely. In contrast, a PLA filament as a heavy eco-friendly material (corn starch) shrinks less, although it is incapable of acetone fumigation [23]. Liquid materials such as photocurable liquid resin and casting wax are utilized for light polymerization and jetting, whereas powder materials are essential for sintering and binder jetting. Therefore, synthetic resin, metal, and clay are used as representative materials in SLS [2,10,35]. Furthermore, thin-layered materials, such as paper, polymer, or metal foil, are used for sheet lamination [6]. 

Silicone as a polymer material is widely applied in the surgical fields [4]. Recently, silicone has been used widely from homes to industries owing to its desirable properties, such as non-ionicity, non-polarity, hydrophobicity, water repellency, thermostability, oxidative stability, frigostability, gas permeability, chemical inertness, environmental friendliness, and non-toxicity [29,36]. The safety profile of silicone enables its practical applications in medicine, pharmaceuticals, cosmetics, and food production, where FDA approval is necessary. Medical silicone appears in the poppets of artificial heart valves, breast implants, plastic prostheses, wound dressing, and catheters [36]. However, external pressures can cause defects in silicone products, which have deficient tear strength and inferior resistance to fatigue. The development of silicone that is more suitable for the human body is in progress through the diversification of various silicone formulations [16,19,37,38]. Because researchers have reported breast-implant-associated anaplastic large-cell lymphoma in certain products, additional research should be conducted regarding this issue [39]. 

Biomaterials in surgeries are designed for in vivo implants or interactions with the body without exhibiting biologic, pharmacological, or histological reactions. Therefore, biomaterials can complement or replace functions of damaged tissues or organs [40]. Biomaterials for the treatment of human diseases imply implant materials that exhibit biocompatibility, i.e., materials that do not cause adverse reactions or toxic responses when exposed to a patient’s tissue, blood, or body fluid [25,28,41]. Neither toxicity nor carcinogenicity should occur during the implantation or degradation of biomaterials in the human body [42]. Additionally, biomaterials should exhibit appropriate mechanical properties without deterioration in physical properties for replacing human tissues [5,43,44]. 

It is important to fabricate a supporting structure comprising regeneration tissues or organs [45]. The supporting structure should facilitate cellular adhesion, proliferation, and differentiation, which can provide an extracellular matrix environment similar to the human body [41]. This structure should exhibit mechanical properties that are suitable for the implant site. Porosity in the supporting structure induces tissue regeneration by allowing cells to infiltrate and nutrients (or oxygen) to flow. Researchers have continued to apply various chemical, physical, and mechanical techniques to this structure [34,37,45,46]. 

**Figure 4:**
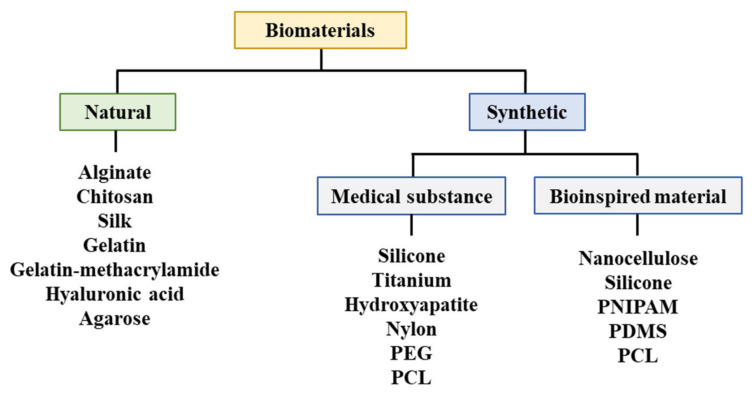
Materials utilized for bioprinting. PEG: Polyethylene glycol, PCL: Polycaprolactone, PNIPAM: Poly(N-isopropylacrylamide), PDMS: Polydimethylsiloxane.

A biodegradable absorbable polymer implies a complete degradation of implants in the human body, which facilitates the adhesion of cells or tissues in regenerative medicine [23]. Additionally, this polymer can be used for drug delivery systems [41,47,48,49]. Therefore, biodegradable polymers are suitable for bioprinting when they comprise metabolites or harmless water-soluble polymer units and exhibit chemical structures that enable hydrolysis. It is propitious to utilize hydrophilic biodegradable polymers that can contain cells and bioactive materials in hydrogels [50,51]. Three-dimensional printing polymeric biomaterials include fibrin, collagen, gelatin, alginate, hyaluronic acid, polyethylene glycol (PEG), Matrigel®, agarose, ABS, polycaprolactone (PCL), PLA, and polylactic-co-glycolic acid (Figure 4) [37,47,50,52,53]. 

According to the available market data for 2020, the price of each different material is as follows: ABS (USD 0.067/g), PLA (USD 0.067/g), resin (USD 50/L), polyadmide (USD 1.4/g), metal powder (USD 0.45/g), wax (USD 200/L), silk fibroin (USD 290/g), alginate (USD 1600/g), chitosan (USD 1.27/g), gelatin-methacrylamide (USD 206/g), hyaluronic acid (USD 60,200/g), agarose (USD 5.83/g), silicone (USD 670/L), titanium (USD 20.3/g), hydroxyapatite (USD 84,200/L), nylon (USD 1.87/g), PEG (USD 1230/g), PCL (USD 9.78/g), nanocellulose (USD 86.9/L), PNIPAM (USD 65.8/g), and PDMS (USD 70/g). Owing to the low filament cost, FDM remains the most popular type of 3D printing hardware.

## 5. Bioprinting Techniques

**Figure 5:**
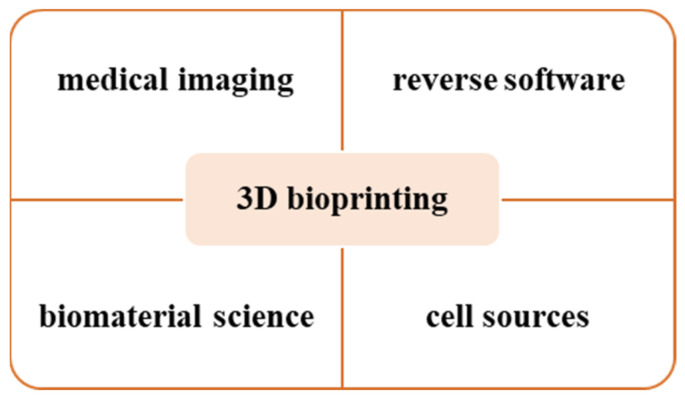
Elements essential for bioprinting.

Bioprinting is a technique to fabricate tissues or organs by stacking 3D-printed cells in the desired shape or pattern (Figure 5) [22]. Bioinks and printing techniques should be considered jointly for bioprinting [27]. As a material applied to bioprinting, bioinks include cells, biomolecules, growth factors, and extracellular matrices [3,37,54,55]. Bioinks provide the physical properties required for processing 3D shapes [34,41,45,47]. Cellular affinity is necessitated in bioinks to provide a favorable environment for cellular proliferation and differentiation [22,47]. For prolonged bioprinting, nutrients and oxygen should be supplied in the cartridge for cellular survival, and cells should be protected from heat and pressure. Human stem cells are isolated and cultured depending on the bioink used [47,56,57,58,59]. Although stem cells transplanted to patients must exhibit growth and differentiation according to their purposes, the size and complexity of tissue-engineered constructs owing to a lack of vasculature typically results in the poor long-term maintenance of mechanical strength [27,60,61].

Typically, extrusion-, inkjet-, and laser-based 3D printers are used for bioprinting in surgeries. Using air or mechanical pressure, extrusion-based printers extrude bioinks from a syringe to produce a 3D cellular structure [4,62]. In extrusion-based printers, bioinks that have a broad range of viscosity and concentrated cells (or cell spheroids) are used. However, these printers with low resolution can negatively affect cellular survival owing to the shear stress on cells. Therefore, the bioinks used in these printers should have shear-thinning properties and maintain the post-printing shape. Pluronic® or biopolymers (agarose and gelatin) can be added to improve the shear-thinning properties of bioink. To maintain a 3D shape during the long period of tissue regeneration, the 3D printout should be post-processed by photopolymerization or chemical crosslinking, which increases the mechanical strength. Materials of thin filaments are appropriate for use as the bioink of extrusion-based printers and include collagen, Matrigel®, photocurable gelatin methacryloyl, collagen methacryloyl, hyaluronic methacrylate, and calcium-alginate gel. Inkjet-based printers, piezoelectrically or thermally, spray 10–50 µm droplets generated from the bioink-containing cells [4]. The cells in the bioink of inkjet-based printers are exposed to heat for 2 μs, which does not much affect cellular survival [57]. The bioink of inkjet-based printers exhibits unfavorable properties of viscosity less than 10 mPa∙s and cellular concentration of less than 106 cells/mL [63]. Therefore, bioinks such as PEG diacrylate and alginate are used in inkjet-based printers [40]. Because these printers do not use nozzles, clogging due to nozzles and cellular exposure to shear stress do not occur [4]. Laser-based printers expel beams of donor ribbons comprising an absorption layer of titanium (or gold) and a bioink layer, which generates and propels droplets [64]. Laser-based printers of 10–100 μm resolution can use bioinks of 1–300 mPa∙s viscosity and 108 cells/mL cellular concentration. Typically, bioinks such as photocurable PEG diacrylate (≒ 20%) and gelatin methacryloyl (≒ 15%) are used in laser-based printers [19,22,32,37,62,65]. Bioinks of poor thermal conductivity can improve cellular survival through temporary heat induced by high energy laser. Research on bioprinting in surgery is underway to conduct clinical trials. 

3D bioprinting techniques can be applied to fabricate all parts of the vulnerable and limited human body based on medical imaging, such as computerized tomography or magnetic resonance imaging (Table 1). Medical imaging enables copies similar to human organs, surgical planning, and education [14,28,42]. In particular, facial reconstruction requires individualized implants. For reconstruction surgery, graft bones are typically harvested from different locations in the body, which increases the risk of pain or infection [2,55,66,67]. Therefore, 3D printing techniques using titanium or PCL as scaffold materials have been applied to the skull and jaw implants [44,68]. Studies regarding the printing of organs for transplantation, such as the kidney, liver, and heart, are in progress [4,6,17,22,56]. All patients with amputated limbs are not fitted well with standardized prostheses. Therefore, printing techniques can be beneficial for complex anatomical structures such as hands [17]. Currently, many standardized breast implants exist [69]. However, these implants can cause negative cosmetic results such as asymmetry [70]. Customized breast implants can be manufactured using 3D printing. Skin grafts are typically used to treat facial disfiguration due to trauma, burn, tumor, or congenital deformity [29]. Skin substitutes for defects cannot function as autologous skin [71]. Optimal skin implants should be durable and resistant to moisture loss and infections to protect irregular wound surfaces. Researchers are investigating techniques for directly printing collagen-based skin on limbs [42,62,72,73], in which collagen-based substrates containing cells are printed in the histologic layers of specific skin types after wounds are analyzed using a scanner. This project is advancing the immediate treatment of wounds and burns using printed skin in a military environment.

The safety of manufactured implants should be secured by ensuring hygiene [79]. For implantations into the human body, requisite safety standards have to be established with regard to quality assurances, adverse events, and clinical trials [32,40,54]. Thereafter, clinical translations should be subjected to regulatory approval for safety, risk, and effectiveness [73]. Furthermore, moral, ethical, and legal concerns should be considered because bioprinting techniques are intended for use in humans [19,40]. A joint committee comprising medical and legal professionals should monitor the exploitation of this technology. Long-term clinical studies are necessary to examine the efficacy, reproducibility, and safety of 3D-printed human body parts [10]. 

## 6. Longitudinal In Vivo NIR Imaging of 3D-Printed Implants

3D bioprinting technologies have the greatest potential in the fabrication of complicated structures. 3D-bioprinted tissues or organs should provide tissue similar structure, biocompatibility, bio-physicochemical microenvironment, etc., to be implanted into the body for tissue regeneration via in vivo integration [20]. To make an accurate estimate of clinical outcomes using 3D-printed implants, reliable preclinical studies should be performed. NIR fluorophore-conjugated scaffolds in 3D printed implants enable the accurate in vivo evaluation of surgical outcomes using NIR imaging.

Traditional histological analysis is one of the main types of preclinical studies to observe tissue growth based on biomaterials at various time points from different animals. This method requires many animals and consequently brings in experimental errors from animal-to-animal variations [80]. Therefore, molecular imaging techniques have been applied to longitudinally visualize the implants in the same animal. Even though technologies such as computed tomography (CT), magnetic resonance imaging (MRI), positron emission tomography (PET), single-photon-emission-computed tomography (SPECT) provide non-invasive volumetric imaging, they are limited in clinics by their high cost, long acquisition times, and non-continuous operation. On the other hand, optical imaging is cost-effective, rapid, easy to use, and can be readily applied to the clinic and surgical setting [81]. In addition, optical imaging can be combined with spectroscopy or other modalities to obtain various sample information. However, optical imaging in the range of visible wavelengths may reduce the reliability of in vivo imaging results due to endogenous tissue autofluorescence. 

Near-infrared (NIR) fluorescence that presents minimal background autofluorescence, reduced light scattering, and low tissue absorption can be a safe and cost-effective optical imaging source with high resolution and high sensitivity [21]. There are many developments of NIR fluorescent imaging technology to fulfill longitudinally in vivo imaging of transplanted cells [82,83,84,85,86,87,88,89,90,91], biomaterials [80,90,92,93,94,95], and tissue growth [80,90,96,97,98,99,100] in the field of regenerative medicine. In a study on the NIR imaging of transplanted cells, Ushiki et al. directly labeled donor bone marrow cells using Alexa Fluor 750 and monitored the homing of transplanted cells shortly after bone marrow transplantation using a whole-body imaging system (IVIS) for diagnosis in the early stages of diseases such as engraftment failure and graft-versus-host disease (GVHD) [87]. Osani et al. verified that the intra-arterial transplantation of bone marrow stem cells (BMSCs) enhanced functional recovery after traumatic brain injury (TBI) by NIR fluorescence imaging. They tracked transplanted BMSCs that were labeled with the QD800 Q-tracker cell labeling kit by IVIS after injection to the injured brain for 4 weeks [86]. For material detection, Gálisová et al. developed a trimodal imaging platform using MRI, NIR fluorescence, and bioluminescence imaging to track transplanted pancreatic islets in vivo. Pancreatic islets labeled with poly(lactic-co-glycolic acid)-based nanoparticles, encapsulated with perfluoro-15-crown-5-ether and NIR fluorescent dye indocyanine green (ICG), were transplanted to rats for 2 weeks and trimodal imaging was performed. Even though the fluorescent imaging of the ICG dye in nanoparticles is unstable under in vivo monitoring, the imaging was very sensitive [94]. Li et al. developed Yb3+ and Ho3+ co-doped fluorapatite (FA:Yb3+/Ho3+) and hydroxyapatite (HAP:Yb3+/Ho3+). The green and red upconversion emission can be generated by NIR (980 nm) excitation, which helps to distinguish the implanted materials from the bone tissue on stained histological sections from in vivo samples [92]. As part of the detection of tissue growth, Cowles et al. applied NIR optical imaging with a bone-specific NIR-targeted probe, IRDye® 800CW Bone Tag^TM^ (800CW BT), in a mouse model in which human mesenchymal stem cells (hMSCs)-seeded gelatin scaffolds were implanted. They longitudinally visualized regions of mineralization of tissue-engineered bone constructs in vivo using the preferential binding of 800CW BT to the mineralized matrix [96]. Hyun et al. developed cartilage-specific NIR fluorophores (C700-OMe and C800-OMe) bound to hyaline, elastic and fibrocartilages and confirmed their specificity in mice [98]. 

**Figure 6:**
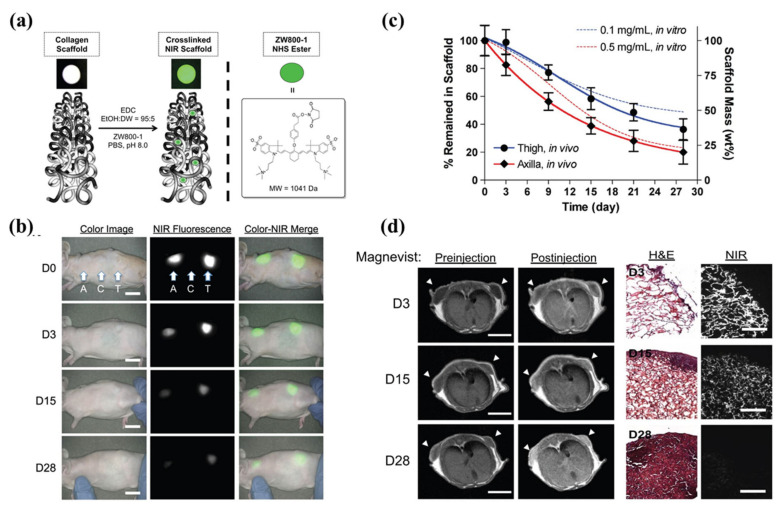
Near-infrared (NIR) fluorophore (ZW800-1)-conjugated collagen scaffold. (**a**) Schematic drawing of NIR scaffold preparation and chemical structure of ZW800-1 NHS ester. (**b**) Optical measurements of scaffold degradation by imaging NIR fluorescence over the skin. A, axilla; C, control; T, thigh, Scale bars = 1 cm. (**c**) Quantification of in vivo scaffold degradation in nude mice. Time course of signal changes in NIR scaffolds in animals (solid lines). In vitro test results were added as dotted lines for comparison. (**d**) Quantification of tissue ingrowth and scaffold degradation. (l) In vivo MR imaging of NIR scaffold implants (axilla part) with and without Magnevist. Arrowheads = implantation sites. Scale bars = 1 cm. (r) H&E and NIR imaging of resected scaffolds from (b, axilla part). Scale bars = 100 μm. Scaffold degradation accompanying tissue ingrowth was compared. Reproduced with permission from [80].

**Figure 7:**
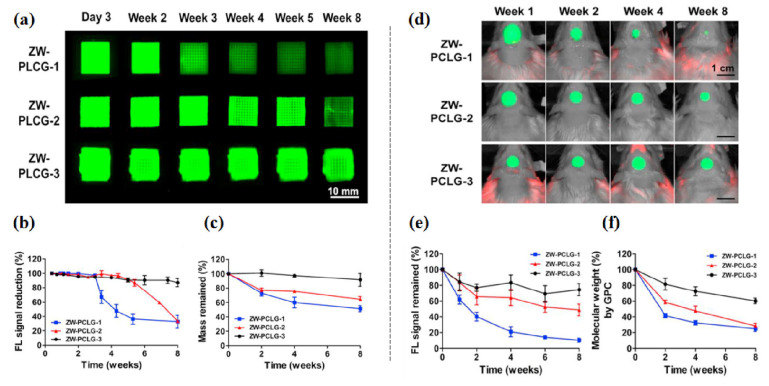
In vitro and in vivo degradation testing of ZW-PCLG copolymers. (**a**–**c**) In vitro degradation test of ZW-PLCGs with different monomer ratios. (**a**) NIR fluorescent images of the printed ZW-PCLG scaffolds under physiological condition. (**b**) Quantification of NIR fluorescence intensity remained in the scaffolds and (**c**) the remaining mass of the ZW-PCLG scaffolds. (**d**–**f**) In vivo noninvasive NIR monitoring for scaffold degradation. (**d**) NIR images of the ZW-PCLG scaffolds in the defected calvarial bone of rats at 1, 2, 4, and 8 weeks post-implantation. (**e**) Quantification of the remaining NIR fluorescent intensity in the ZW-PCLG scaffolds and (**f**) molecular weight changes measured by gel permeation chromatography (GPC). Reproduced with permission from [90].

**Figure 8:**
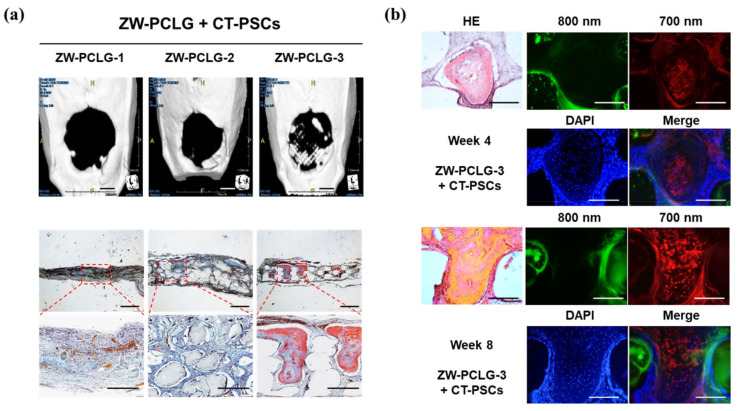
Computed tomography (CT) and histological examinations of the CT-PSCs and PCLG scaffolds. (**a**) New bone formation in the calvarial bone defect was examined by (top) CT (scale bar = 2 mm) and (bottom) modified Tetrachrome staining (scale bar = 1 mm). (**b**) High-power images to confirm hPSCs within the bone matrix in ZW-PCLG-3 + CT-PSCs. Green pseudocolor at 800 nm indicates the remaining PCLG scaffold, red pseudocolor at 700 nm indicates hPSCs, and the blue color indicates DAPI-stained nuclei. Scale bar = 500 μm. Reproduced with permission from [90].

To date, only a few studies have been carried out in 3D constructs [80,90,91,95,97]. In regard to biomaterials/tissue imaging in 3D constructs, Kim et al. developed a NIR fluorophore-conjugated small intestinal submucosa-based scaffold and gelatin-based scaffold to observe their degradation behavior and the tissue formation in them [80,95] (Figure 6). Scaffolds were conjugated with NIR fluorophores such as zwitterionic heptamethine indocyanine (ZW800-1) or meso-brominated NIR fluorescence pentamethine cyanine, and successfully tracked the biodegradation pattern and rate of scaffolds using real-time noninvasive NIR imaging in the same nude mouse. In addition, they proved scaffold degradation along with tissue infiltration in mice using MRI and NIR microscopy. Recently, Park et al. reported a dual-channel NIR fluorescence imaging technique to monitor brain tissue ingrowth and NIR hydrogel degradation simultaneously [97]. Enzymatically crosslinked gelatin-hyaluronic acid hydrogel was labeled with 800 nm fluorophore (ZW800-3a), while the regenerated brain was tracked using 700 nm brain-specific contrast agent (Ox1). Besides the enzymatic degradable scaffolds, Kim et al. employed ZW800-1 on synthetic polymers, PCL-ran-PLLA-ran-PGA (PCLG copolymers), (ZW-PCLG) with three different degradation rates to confirm the fate of implanted scaffolds depending on degradation rate [90]. They fabricated NIR fluorophore-conjugated scaffolds by a 3D-printing technique in order to obtain reliable results by precisely controlled structure. In both in vitro and in vivo studies (Figure 7), the suggested NIR imaging system could noninvasively monitor the scaffold-degradable behavior according to the intended degradation rate. Furthermore, they achieved simultaneous monitoring of scaffold and transplanted stem cells under the same 3D printed construct using NIR fluorescence imaging system. Human placental stem cells (hPSCs) were labeled with 700 nm emitting lipophilic pentamethine, CTNF127, (CT-PSCs) while the PCLG copolymers were labeled with 800 nm fluorophore, ZW800-1, (ZW-PCLG). Cells and biomaterial labeled by NIR fluorophore were 3D-printed in one structure and implanted into the rat calvarial bone defect model. Figure 8 illustrates tracing transplanted cells and scaffold without specific staining using dual-channel NIR fluorescence microscopy after the construct retrieval. Through this study, they found that the slowly degradable biomaterial with stem cells showed the highest new bone formation compared to the rapidly degradable biomaterial. To the best of our knowledge, previously introduced technologies have not solved the monitoring of both cell and scaffold at the same time, even though scaffold and cells coexist and interact with each other inside engineered tissue. In this situation, this study is very meaningful; however, the simultaneously quantifiable dual-monitoring of cell and scaffold in vivo using NIR-based noninvasive monitoring method should be investigated for easy application. 

These studies have proved that NIR fluorescence imaging is valuable to detect biomaterial degradation in vivo without having to sacrifice animals. However, the noninvasive applications using NIR fluorescence imaging are limited to monitoring only in superficial tissues and organs (e.g., skin, muscle, cartilage, bone, and adipose tissue) due to inherent optical scattering. In order to monitor 3D structures implanted in deeper tissues or organs, the minimally invasive endoscope or invasive surgical procedures are required. Otherwise, multimodal imaging that combines NIR imaging with other imaging modalities such as MRI, mesoscale imaging, and microCT could be useful in non-invasive monitoring of tissue regeneration processes based on a 3D (printed) construct. 

## 7. Summary and Conclusions

This concise review provides an overview of 3D printing for implant fabrication and the application of non-invasive monitoring methods using in vivo NIR fluorescence imaging for 3D printed constructs. 

We have categorized 3D printing hardware according to their discriminative process, although innovative products are under development. Those types are extrusion, direct energy deposition, lamination, light polymerization, jetting, sintering, and binder jetting. Modeling prepared in 3D printing software is followed by programmatic slicing to create G-codes, which 3D printers can understand. Extrusion, direct energy deposition, and lamination are based on solid materials, whereas light polymerization and jetting are based on liquid materials. However, powder is used for sintering and binder jetting. 

Bioprinting typically involves extrusions, inkjets, and laser-based 3D printers. The biomaterials used are inert substances designed for implantation or in vivo interaction, which complement or replace the function of living tissues or organs. As a material applied to bioprinting, bioink includes cells, biomolecules, growth factors, and extracellular matrices. Furthermore, bioprinting requires the consideration of moral, ethical, and legal concerns. 

Imaging 3D constructs at greater than a millimeter scale in the body is important and difficult in the development of a 3D structure by 3D bioprinting. Among various imaging modalities, the NIR fluorescence imaging method offers relatively high spatial and temporal resolution with deeper tissue penetration, minimum tissue absorption, and autofluorescence. In the last section, we introduced several studies that achieved monitoring of biomaterials, cells, and/or tissue formation via 3D construct using NIR-based imaging system. 

In conclusion, 3D printing techniques equipped with high-performance hardware and software have been created in various areas. 3D printing techniques may be especially applicable to the vulnerable and limited domain of biomedical research and healthcare. Besides fabrication, evaluation of a 3D-printed construct is very important. An NIR imaging system could accelerate the development of 3D printing technology in clinics by providing precise and easy analysis methods for materials, cells, and/or tissue ingrowth. Physicians and researchers in the surgical field should understand and give attention to its enabling technologies as well as the basics of 3D printing. In this context, this review can help to achieve successful clinical outcomes via 3D printing technology.

## Figures and Tables

**Table 1:** Clinical applications of 3D printing in surgical fields.

Study	Year	Clinical Application	Material
Gerstle [67]	2014	Surgical planning, training, and patient education	3D modeling software
Klammert [74]	2010	Bone and craniomaxillofacial reconstruction	Polycaprolactone Polyetherketoneketone Adipose-derived stem cell
Ziegler-Graham [75]	2008	Upper extremity and hand prosthetics	Thermoplastic Aluminum Stainless steel
Lu [64]	2019	Lower extremity and foot prosthetics	Titanium ß-TCP bioceramic
Visser [76]	2012	Nose, ear, and cartilage reconstruction	Injectable gel Collagen Silicone Chondrocyte
Rengier [32]	2010	Breast reconstruction	Absorbable biologic matrix Silicone
Hann [77]	2019	Cardiac and vascular reconstruction	Gelatin methacryloyl Stem cell
Debels [78]	2014	Skin	Collagen Hydrogel biopolymer composite Fibrin Stem cell

## References

[1] Murphy S.V., De Coppi P., Atala A. (2019). Opportunities and challenges of translational 3D bioprinting. Nat. Biomed. Eng..

[2] Lin K., Sheikh R., Romanazzo S., Roohani I. (2019). 3D printing of bioceramic scaffolds—barriers to the clinical translation: From promise to reality, and future perspectives. Materials.

[3] Ratinam R., Quayle M., Crock J., Lazarus M., Fogg Q., McMenamin P. (2019). Challenges in creating dissectible anatomical 3D prints for surgical teaching. J. Anat..

[4] Kryou C., Leva V., Chatzipetrou M., Zergioti I. (2019). Bioprinting for liver transplantation. Bioengineering.

[5] Papaioannou T.G., Manolesou D., Dimakakos E., Tsoucalas G., Vavuranakis M., Tousoulis D. (2019). 3D bioprinting methods and techniques: Applications on artificial blood vessel fabrication. Acta Cardiol. Sin..

[6] Pietrabissa A., Marconi S., Negrello E., Mauri V., Peri A., Pugliese L., Marone E.M., Auricchio F. (2020). An overview on 3D printing for abdominal surgery. Surg. Endosc..

[7] Tack P., Victor J., Gemmel P., Annemans L. (2016). 3D-printing techniques in a medical setting: A systematic literature review. Biomed. Eng. Online.

[8] Ruiters S., Mombaerts I. (2019). Applications of three-dimensional printing in orbital diseases and disorders. Curr. Opin. Ophthalmol..

[9] Tong Y., Kaplan D.J., Spivak J.M., Bendo J.A. (2019). Three-dimensional printing in spine surgery: A review of current applications. Spine J..

[10] Liaw C.-Y., Guvendiren M. (2017). Current and emerging applications of 3D printing in medicine. Biofabrication.

[11] Galliger Z., Vogt C.D., Panoskaltsis-Mortari A. (2019). 3D bioprinting for lungs and hollow organs. Transl. Res..

[12] Smith B., Dasgupta P. (2019). 3D printing technology and its role in urological training. World J. Urol..

[13] Jiang M., Chen G., Coles-Black J., Chuen J., Hardidge A. (2020). Three-dimensional printing in orthopaedic preoperative planning improves intraoperative metrics: A systematic review. ANZ J. Surg..

[14] Velázquez J.S., Cavas F., Bolarín J.M., Alió J.L. (2020). 3D printed personalized corneal models as a tool for improving patient’s knowledge of an asymmetric disease. Symmetry.

[15] Pugliese L., Marconi S., Negrello E., Mauri V., Peri A., Gallo V., Auricchio F., Pietrabissa A. (2018). The clinical use of 3D printing in surgery. Updates Surg..

[16] Mitsouras D., Liacouras P., Imanzadeh A., Giannopoulos A.A., Cai T., Kumamaru K.K., George E., Wake N., Caterson E.J., Pomahac B. (2015). Medical 3D printing for the radiologist. Radiographics.

[17] Aimar A., Palermo A., Innocenti B. (2019). The role of 3D printing in medical applications: A state of the art. J. Healthc. Eng..

[18] Parthasarathy J., Krishnamurthy R., Ostendorf A., Shinoka T., Krishnamurthy R. (2020). 3D printing with MRI in pediatric applications. J. Magn. Reson. Imaging.

[19] Tejo-Otero A., Buj-Corral I., Fenollosa-Artés F. (2020). 3D printing in medicine for preoperative surgical planning: A review. Ann. Biomed. Eng..

[20] Cui H., Nowicki M., Fisher J.P., Zhang L.G. (2017). 3D bioprinting for organ regeneration. Adv. Healthc. Mater..

[21] Choi H.S., Gibbs S.L., Lee J.H., Kim S.H., Ashitate Y., Liu F., Hyun H., Park G., Xie Y., Bae S. (2013). Targeted zwitterionic near-infrared fluorophores for improved optical imaging. Nat. Biotechnol..

[22] Zhang S., Wang H. (2019). Current Progress in 3D Bioprinting of Tissue Analogs. Slas Technol.

[23] Mazzanti V., Malagutti L., Mollica F. (2019). FDM 3D printing of polymers containing natural fillers: A review of their mechanical properties. Polymers.

[24] Chen M.Y., Skewes J., Desselle M., Wong C., Woodruff M.A., Dasgupta P., Rukin N.J. (2020). Current applications of three-dimensional printing in urology. Bju Int..

[25] Zhang J.M., Ji Q., Duan H. (2019). Three-dimensional printed devices in droplet microfluidics. Micromachines.

[26] Han T., Kundu S., Nag A., Xu Y. (2019). 3D printed sensors for biomedical applications: A Review. Sensors.

[27] Jessop Z.M., Al-Sabah A., Gardiner M.D., Combellack E., Hawkins K., Whitaker I.S. (2017). 3D bioprinting for reconstructive surgery: Principles, applications and challenges. J. Plast. Reconstr. Aesthet. Surg..

[28] Mandrycky C., Wang Z., Kim K., Kim D.H. (2016). 3D bioprinting for engineering complex tissues. Biotechnol. Adv..

[29] Mussi E., Furferi R., Volpe Y., Facchini F., McGreevy K.S., Uccheddu F. (2019). Ear reconstruction simulation: From handcrafting to 3D printing. Bioengineering.

[30] Virzi A., Muller C.O., Marret J.B., Mille E., Berteloot L., Grevent D., Boddaert N., Gori P., Sarnacki S., Bloch I. (2020). Comprehensive review of 3D segmentation software tools for MRI usable for pelvic surgery planning. J. Digit. Imaging.

[31] Blake C., Birch S., Brandao J. (2019). Medical three-dimensional printing in zoological medicine. Vet. Clin. N. Am. Exot. Anim. Pr..

[32] Leberfinger A.N., Dinda S., Wu Y., Koduru S.V., Ozbolat V., Ravnic D.J., Ozbolat I.T. (2019). Bioprinting functional tissues. Acta Biomater..

[33] Araujo M.R.P., Sa-Barreto L.L., Gratieri T., Gelfuso G.M., Cunha-Filho M. (2019). The digital pharmacies era: How 3D printing technology using fused deposition modeling can become a reality. Pharmaceutics.

[34] Hoque M.E., Chuan Y.L., Pashby I. (2012). Extrusion based rapid prototyping technique: An advanced platform for tissue engineering scaffold fabrication. Biopolymers.

[35] Rengier F., Mehndiratta A., von Tengg-Kobligk H., Zechmann C.M., Unterhinninghofen R., Kauczor H.U., Giesel F.L. (2010). 3D printing based on imaging data: Review of medical applications. Int. J. Comput. Assist. Radiol. Surg..

[36] Kim I.S. (2018). Augmentation rhinoplasty using silicone implants. Facial Plast. Surg. Clin. N. Am..

[37] Huang Y., Zhang X.F., Gao G., Yonezawa T., Cui X. (2017). 3D bioprinting and the current applications in tissue engineering. Biotechnol. J..

[38] Roh S., Parekh D.P., Bharti B., Stoyanov S.D., Velev O.D. (2017). 3D Printing by multiphase silicone/water capillary inks. Adv. Mater..

[39] Mempin M., Hu H., Chowdhury D., Deva A., Vickery K. (2018). The A, B and C’s of silicone breast implants: Anaplastic large cell lymphoma, biofilm and capsular contracture. Materials.

[40] Angelopoulos I., Allenby M.C., Lim M., Zamorano M. (2020). Engineering inkjet bioprinting processes toward translational therapies. Biotechnol. Bioeng..

[41] Patel D.K., Lim K.T. (2019). Biomimetic polymer-based engineered scaffolds for improved stem cell function. Materials.

[42] Bauermeister A.J., Zuriarrain A., Newman M.I. (2016). Three-dimensional printing in plastic and reconstructive surgery: A systematic review. Ann. Plast. Surg..

[43] Parak A., Pradeep P., du Toit L.C., Kumar P., Choonara Y.E., Pillay V. (2019). Functionalizing bioinks for 3D bioprinting applications. Drug Discov. Today.

[44] Ostrovidov S., Salehi S., Costantini M., Suthiwanich K., Ebrahimi M., Sadeghian R.B., Fujie T., Shi X., Cannata S., Gargioli C. (2019). 3D bioprinting in skeletal muscle tissue engineering. Small.

[45] Salerno A., Cesarelli G., Pedram P., Netti P.A. (2019). Modular strategies to build cell-free and cell-laden scaffolds towards bioengineered tissues and organs. J. Clin. Med..

[46] Hippler M., Lemma E.D., Bertels S., Blasco E., Barner-Kowollik C., Wegener M., Bastmeyer M. (2019). 3D Scaffolds to Study Basic Cell Biology. Adv. Mater..

[47] Dzobo K., Motaung K., Adesida A. (2019). Recent trends in decellularized extracellular matrix bioinks for 3D printing: An Updated Review. Int. J. Mol. Sci..

[48] Prendergast M.E., Burdick J.A. (2020). Recent advances in enabling technologies in 3D printing for precision medicine. Adv. Mater..

[49] Algahtani M.S., Mohammed A.A., Ahmad J. (2018). Extrusion-based 3D printing for pharmaceuticals: Contemporary research and applications. Curr. Pharm. Des..

[50] Liu F., Chen Q., Liu C., Ao Q., Tian X., Fan J., Tong H., Wang X. (2018). Natural polymers for organ 3D bioprinting. Polymers.

[51] Xu C., Dai G., Hong Y. (2019). Recent advances in high-strength and elastic hydrogels for 3D printing in biomedical applications. Acta Biomater..

[52] Marques C.F., Diogo G.S., Pina S., Oliveira J.M., Silva T.H., Reis R.L. (2019). Collagen-based bioinks for hard tissue engineering applications: A comprehensive review. J. Mater. Sci. Mater. Med..

[53] Li S., Tian X., Fan J., Tong H., Ao Q., Wang X. (2019). Chitosans for tissue repair and organ three-dimensional (3D) bioprinting. Micromachines.

[54] Valot L., Martinez J., Mehdi A., Subra G. (2019). Chemical insights into bioinks for 3D printing. Chem. Soc. Rev..

[55] Wang Q., Han G., Yan S., Zhang Q. (2019). 3D printing of silk fibroin for biomedical applications. Materials.

[56] Eswaramoorthy S.D., Ramakrishna S., Rath S.N. (2019). Recent advances in three-dimensional bioprinting of stem cells. J. Tissue Eng. Regen. Med..

[57] Gao G., Schilling A.F., Hubbell K., Yonezawa T., Truong D., Hong Y., Dai G., Cui X. (2015). Improved properties of bone and cartilage tissue from 3D inkjet-bioprinted human mesenchymal stem cells by simultaneous deposition and photocrosslinking in PEG-GelMA. Biotechnol. Lett..

[58] Salaris F., Rosa A. (2019). Construction of 3D in vitro models by bioprinting human pluripotent stem cells: Challenges and opportunities. Brain Res..

[59] Romanazzo S., Nemec S., Roohani I. (2019). iPSC Bioprinting: Where are we at?. Materials.

[60] Wang X., Liu C. (2018). 3D bioprinting of adipose-derived stem cells for organ manufacturing. Adv. Exp. Med. Biol..

[61] Ong C.S., Yesantharao P., Huang C.Y., Mattson G., Boktor J., Fukunishi T., Zhang H., Hibino N. (2018). 3D bioprinting using stem cells. Pediatr Res..

[62] Kacarevic Z.P., Rider P.M., Alkildani S., Retnasingh S., Smeets R., Jung O., Ivanisevic Z., Barbeck M. (2018). An introduction to 3D bioprinting: Possibilities, challenges and future aspects. Materials.

[63] Hoch E., Hirth T., Tovar G.E.M., Borchers K. (2013). Chemical tailoring of gelatin to adjust its chemical and physical properties for functional bioprinting. J. Mater. Chem. B.

[64] Heinrich M.A., Liu W., Jimenez A., Yang J., Akpek A., Liu X., Pi Q., Mu X., Hu N., Schiffelers R.M. (2019). 3D bioprinting: From benches to translational applications. Small.

[65] Matai I., Kaur G., Seyedsalehi A., McClinton A., Laurencin C.T. (2020). Progress in 3D bioprinting technology for tissue/organ regenerative engineering. Biomaterials.

[66] Ballard D.H., Tappa K., Boyer C.J., Jammalamadaka U., Hemmanur K., Weisman J.A., Alexander J.S., Mills D.K., Woodard P.K. (2019). Antibiotics in 3D-printed implants, instruments and materials: Benefits, challenges and future directions. J. 3d Print. Med..

[67] Lu Y., Chen G., Long Z., Li M., Ji C., Wang F., Li H., Lu J., Wang Z., Li J. (2019). Novel 3D-printed prosthetic composite for reconstruction of massive bone defects in lower extremities after malignant tumor resection. J. Bone Oncol..

[68] Midha S., Dalela M., Sybil D., Patra P., Mohanty S. (2019). Advances in three-dimensional bioprinting of bone: Progress and challenges. J. Tissue Eng. Regen. Med..

[69] Gerstle T.L., Ibrahim A.M., Kim P.S., Lee B.T., Lin S.J. (2014). A plastic surgery application in evolution: Three-dimensional printing. Plast. Reconstr. Surg..

[70] Cleversey C., Robinson M., Willerth S.M. (2019). 3D printing breast tissue models: A review of past work and directions for future work. Micromachines.

[71] Varkey M., Visscher D.O., van Zuijlen P.P.M., Atala A., Yoo J.J. (2019). Skin bioprinting: The future of burn wound reconstruction?. Burn. Trauma.

[72] Correia Carreira S., Begum R., Perriman A.W. (2020). 3D bioprinting: The emergence of programmable biodesign. Adv. Healthc. Mater..

[73] Singh S., Choudhury D., Yu F., Mironov V., Naing M.W. (2020). In situ bioprinting - Bioprinting from benchside to bedside?. Acta Biomater..

[74] Klammert U., Gbureck U., Vorndran E., Rodiger J., Meyer-Marcotty P., Kubler A.C. (2010). 3D powder printed calcium phosphate implants for reconstruction of cranial and maxillofacial defects. J. Cranio-Maxillofac. Surg..

[75] Ziegler-Graham K., MacKenzie E.J., Ephraim P.L., Travison T.G., Brookmeyer R. (2008). Estimating the prevalence of limb loss in the United States: 2005 to 2050. Arch. Phys. Med. Rehabil..

[76] Visser J., Melchels F.P., Jeon J.E., van Bussel E.M., Kimpton L.S., Byrne H.M., Dhert W.J., Dalton P.D., Hutmacher D.W., Malda J. (2015). Reinforcement of hydrogels using three-dimensionally printed microfibres. Nat. Commun..

[77] Hann S.Y., Cui H., Esworthy T., Miao S., Zhou X., Lee S.J., Fisher J.P., Zhang L.G. (2019). Recent advances in 3D printing: Vascular network for tissue and organ regeneration. Transl. Res..

[78] Debels H., Hamdi M., Abberton K., Morrison W. (2015). Dermal matrices and bioengineered skin substitutes: A critical review of current options. Plast. Reconstr. Surg. Glob. Open..

[79] Sheha E.D., Gandhi S.D., Colman M.W. (2019). 3D printing in spine surgery. Ann. Transl. Med..

[80] Kim S.H., Lee J.H., Hyun H., Ashitate Y., Park G., Robichaud K., Lunsford E., Lee S.J., Khang G., Choi H.S. (2013). Near-infrared fluorescence imaging for noninvasive trafficking of scaffold degradation. Sci. Rep..

[81] Lee J.H., Park G., Hong G.H., Choi J., Choi H.S. (2012). Design considerations for targeted optical contrast agents. Quant. Imaging Med. Surg..

[82] Kim S.H., Park G., Hyun H., Lee J.H., Ashitate Y., Choi J., Hong G.H., Owens E.A., Henary M., Choi H.S. (2013). Near-infrared lipophilic fluorophores for tracing tissue growth. Biomed. Mater..

[83] Su X., Shen Y., Weintraub N.L., Tang Y. (2019). Imaging and tracking stem cell engraftment in ischemic hearts by near-infrared fluorescent protein (iRFP) labeling. Methods Mol. Biol..

[84] Liu H., Tan Y., Xie L., Yang L., Zhao J., Bai J., Huang P., Zhan W., Wan Q., Zou C. (2016). Self-assembled dual-modality contrast agents for non-invasive stem cell tracking via near-infrared fluorescence and magnetic resonance imaging. J. Colloid Interface Sci..

[85] Hayashi K., Nakamura M., Ishimura K. (2012). Silica–porphyrin hybrid nanotubes for in vivo cell tracking by near-infrared fluorescence imaging. Chem. Commun..

[86] Osanai T., Kuroda S., Sugiyama T., Kawabori M., Ito M., Shichinohe H., Kuge Y., Houkin K., Tamaki N., Iwasaki Y. (2012). Therapeutic effects of intra-arterial delivery of bone marrow stromal cells in traumatic brain injury of rats—In vivo cell tracking study by near-infrared fluorescence imaging. Neurosurgery.

[87] Ushiki T., Kizaka-Kondoh S., Ashihara E., Tanaka S., Masuko M., Hirai H., Kimura S., Aizawa Y., Maekawa T., Hiraoka M. (2010). Noninvasive tracking of donor cell homing by near-infrared fluorescence imaging shortly after bone marrow transplantation. PLoS ONE.

[88] Christian D.A., Garbuzenko O.B., Minko T., Discher D.E. (2010). Polymer vesicles with a red cell-like surface charge: Microvascular imaging and in vivo tracking with near-infrared fluorescence. Macromol. Rapid Commun..

[89] Christian N.A., Benencia F., Milone M.C., Li G., Frail P.R., Therien M.J., Coukos G., Hammer D.A. (2009). In vivo dendritic cell tracking using fluorescence lifetime imaging and near-infrared-emissive polymersomes. Mol. Imaging Biol..

[90] Kim S.H., Park J.H., Kwon J.S., Cho J.G., Park K.G., Park C.H., Yoo J.J., Atala A., Choi H.S., Kim M.S. (2020). NIR fluorescence for monitoring in vivo scaffold degradation along with stem cell tracking in bone tissue engineering. Biomaterials.

[91] Chikate T.R., Tang L. (2019). Tracking and Imaging of Transplanted Stem Cells in Animals. Methods Mol. Biol..

[92] Li X., Chen H. (2016). Yb3+/Ho3+ co-doped apatite upconversion nanoparticles to distinguish implanted material from bone tissue. ACS Appl. Mater. Interfaces.

[93] Li X., Bottini M., Zhang L., Zhang S., Chen J., Zhang T., Liu L., Rosato N., Ma X., Shi X. (2018). Core–satellite nanomedicines for in vivo real-time monitoring of enzyme-activatable drug release by fluorescence and photoacoustic dual-modal imaging. ACS Nano.

[94] Gálisová A., Herynek V., Swider E., Sticova E., Patikova A., Kosinova L. (2019). A Trimodal imaging platform for tracking viable transplanted pancreatic islets in vivo: F-19 MR, fluorescence, and bioluminescence imaging. Mol. Imaging Biol..

[95] Owens E.A., Hyun H., Kim S.H., Lee J.H., Park G., Ashitate Y., Choi J., Hong G.H., Alyabyev S., Lee S.J. (2013). Highly charged cyanine fluorophores for trafficking scaffold degradation. Biomed. Mater..

[96] Cowles E.A., Kovar J.L., Curtis E.T., Xu H., Othman S.F. (2013). Near-infrared optical imaging for monitoring the regeneration of osteogenic tissue-engineered constructs. Biores. Open Access.

[97] Park G.K., Kim S.-H., Kim K., Das P., Kim B.-G., Kashiwagi S., Choi H.S., Hwang N.S. (2019). Dual-channel fluorescence imaging of hydrogel degradation and tissue regeneration in the brain. Theranostics.

[98] Hyun H., Owens E.A., Wada H., Levitz A., Park G., Park M.H., Frangioni J.V., Henary M., Choi H.S. (2015). Cartilage-specific near-infrared fluorophores for biomedical imaging. Angew. Chem..

[99] Hyun H., Wada H., Bao K., Gravier J., Yadav Y., Laramie M., Henary M., Frangioni J.V., Choi H.S. (2014). Phosphonated near-infrared fluorophores for biomedical imaging of bone. Angew. Chem..

[100] Lim W., Kim B., Jo G., Yang D.H., Park M.H., Hyun H. (2020). Bioluminescence and near-infrared fluorescence imaging for detection of metastatic bone tumors. Lasers Med Sci..

